# Effect of Reformer Spring Resistance Modifications on Core Muscle Activity During Basic Core Muscle Exercises

**DOI:** 10.3390/healthcare12232447

**Published:** 2024-12-05

**Authors:** Hee-Jeong Kim, Jung-Ha Sung, Jae-Kyun Ryu, Hyun-Chul Jung, Junsig Wang

**Affiliations:** 1 Graduate School of Physical Education, Kyung Hee University, Yongin 17104, Republic of Korea; heejeong@khu.ac.kr (H.-J.K.); jung.ha@khu.ac.kr (J.-H.S.); ryu69kor@khu.ac.kr (J.-K.R.); jhc@khu.ac.kr (H.-C.J.); 2 Department of Sports Coaching, College of Physical Education, Kyung Hee University, Yongin 17104, Republic of Korea; 3 Department of Sports Medicine, College of Physical Education, Kyung Hee University, Yongin 17104, Republic of Korea

**Keywords:** core muscles, trunk stability, reformer, electromyography, EMG, unstable surface

## Abstract

Background: Core muscles serve as a central group within the functional kinetic chain, stabilizing the spine during movement. The Reformer is one of the most popular and primary pieces used in Pilates core exercises, requiring effective control of core muscles. The purpose of this study was to investigate the effect of Reformer spring resistance on core muscle activity. Method: 18 healthy adult females (age: 29.83 ± 4.22 years, body height: 164.98 ± 4.14 cm, body mass: 53.67 ± 5.32 kg) participated in this study. Surface electromyography was recorded from six muscles: rectus abdominis, internal oblique, erector spinae, multifidus, rectus femoris, and biceps femoris during core exercises. Participants performed three core exercises (hip roll, knee-off, and elephant) under three different Reformer spring conditions (fixed platform, platform connected to springs with moderate resistance, platform connected to springs with low resistance). Results: During the hip roll exercise, mean muscle activity of the rectus abdominis, erector spinae, multifidus, and biceps femoris was significantly increased on the low-resistance spring platform compared to the moderate-resistance platform and fixed platform conditions (*p* < 0.001). During the knee-off exercise, mean muscle activity of the rectus abdominis, internal oblique, erector spinae, and multifidus significantly increased on the low-resistance platform compared to the fixed platform (*p* < 0.001). Furthermore, during the elephant exercise, mean muscle activity of the rectus abdominis, internal oblique, and rectus femoris significantly increased on the low-resistance spring platform compared to the fixed platform (*p* < 0.001). Conclusions: These findings suggest the unstable platform caused by the Reformer spring modification impacts core muscle activity during basic core exercises. Therefore, when designing core exercise programs using the Reformer, platform instability should be considered a key factor for rehabilitation and core stability.

## 1. Introduction

Core muscles include the abdominal muscles in the front, the glutes and the muscles around the spine in the back, the diaphragm at the top, and the pelvic floor and hip muscles at the bottom. The core muscles form the central part of the torso, serve to stabilize the spine, and play a crucial role in the functional kinetic chain during movement [1]. Also, core muscle strength is a basis for maintaining postural stability, injury prevention, pain reduction, and physical performance. Recently, a variety of training methods have been introduced and emphasized in order to prevent lumbar and musculoskeletal disorders, and to enhance athletic performance [2,3].

Pilates is a functional exercise focused on improving core muscle strength and stability [4,5,6]. Previous studies have indicated that Pilates has positive effects on strengthening core muscles and increasing spinal flexibility as a functional exercise based on the stabilization of the proximal region [7,8]. Other studies on Pilates have also suggested its effectiveness in strengthening core muscles and alleviating lower back pain, as well as benefiting overall physical and mental health [6,9,10,11,12]. Moreover, recent clinical experts recommend Pilates as a therapeutic exercise method for rehabilitating low back pain and scoliosis [4,13,14]. Thus, Pilates exercises appear to be one of the effective training methods for strengthening core muscles and improving spinal stabilization.

Among the various types of equipment, the Reformer is one of the most popular and primary pieces used in core exercises, requiring effective control of core muscles [15,16]. It consists of a sliding carriage, springs for resistance, and an adjustable foot bar. Accordingly, the key principle of the core exercises on the Reformer is spring resistance modification according to skill levels and physical conditions, since the spring resistance creates an unstable platform that is needed to control core muscle activity and strength [17]. Previous studies have reported that Reformer Pilates exercises increased core muscle activity compared to exercises performed on a flat mat [18]. Furthermore, another study found that core muscle exercises on unstable surfaces increased core muscle activity responsible for spine stabilization prior to the initiation of movement [19,20], and core muscles were activated approximately 30 milliseconds before the onset of movement for various postural adjustments [21]. These findings support the idea that surface stability impacts core muscle activity for postural stabilization during core exercises. However, few studies explained how varying spring resistance (creating unstable platforms) on a Pilates Reformer influences core muscle activity during core muscle exercises.

Therefore, the purpose of this study was to investigate the effect of Reformer spring resistance on core muscle activity. Specifically, this study aims to determine how surface instability created by varying Reformer spring resistance impacts the engagement of core muscle activity associated with core stability. We hypothesized that core muscle activity would be increased on a low-resistance spring platform compared to a moderate-resistance spring and fixed platforms during Reformer core muscle exercises. A recent study investigated the effect of low vs. heavy spring resistance [14], but more studies are needed to better understand how different levels of spring resistance and surface stability impact core muscle activity during core exercises. A less stable surface requires greater muscle activity to maintain balance and posture. This study has the potential to significantly impact rehabilitation and fitness programs. By understanding how different levels of spring resistance affect core muscle engagement, trainers and therapists could design more personalized exercise programs aimed at improving core strength and stability.

## 2. Materials and Methods

### 2.1. Participants

Eighteen healthy females with a mean (±SD) age of 29.83 (±4.22) years, height of 164.98 (±4.14) cm, and mass of 53.67 ± 5.32 kg were recruited in this study. G*Power 3.1 was utilized to calculate a required sample size of 18 based on our pilot data, aiming for a minimum estimated effect size of 0.30, with an alpha error probability of 0.05 and a power of 0.80. Participants have been practicing Pilates exercises consistently for at least two years, engaging in Pilates sessions for two hours per week. Before participating in the study, each participant read and signed an informed consent form that was approved by the university’s institutional review board (IRB ID: KHGIRB-23-163). Then, we asked the participants about their eligibility and excluded them if they had a history of low back disorders, joint injuries, surgeries in the past six months, or any other disabilities that would affect their ability to perform core exercises.

### 2.2. Experimental Procedure

**Figure 1:**
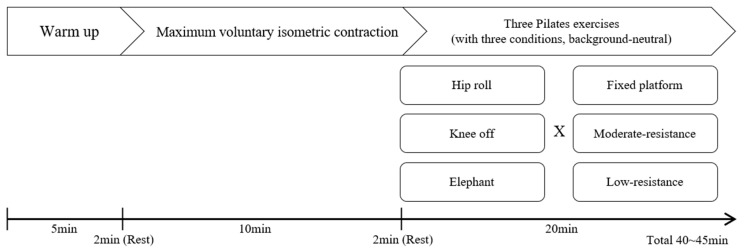
Experimental procedures.

The participants performed three core exercises under three Reformer spring conditions (fixed platform, platform connected to springs with moderate resistance, platform connected to springs with low resistance). Prior to testing, dynamic stretching exercises and pre-exercises were performed to provide part of the warm-up routine and to confirm participants’ understanding of Pilates breathing techniques for core muscle exercise [22]. Then, two trials of maximum voluntary isometric contraction (MVIC) were conducted for core muscles through a manual muscle test. The participants were asked to perform five different isometric exercises for the MVIC trials. For the IO MVIC, participants were positioned in a supine lying and instructed to flex and rotate their trunk against contralateral manual resistance with their hands placed across their shoulders [23,24]. The RA MVIC was assessed during trunk flexion in a sitting position with straight knees, while bilateral manual resistance was applied to both shoulders [23,24]. The MVIC of the ES and MU was measured during trunk extension in a prone position, with manual resistance applied to the posterior shoulders [23,25]. The MVIC for the BF and RF was measured during knee flexion and extension, respectively, with participants in the prone position and their knees flexed at 90 degrees. Manual resistance was applied to the posterior ankle for BF and to the anterior ankle for RF [26]. After the MVIC trials, they performed three core exercises under three Reformer spring conditions (fixed platform, platform connected to springs with moderate resistance, platform connected to springs with low resistance). Each movement was repeated three times, and the average EMG values were used for statistical analyses. We randomized the order of the experimental conditions and conducted a total of nine trials (three exercises × three Reformer spring resistance conditions), (Figure 1). All participants were allowed to rest as needed between conditions, with a minimum break of two minutes. All tests were conducted by two researchers who were experienced with the Reformer apparatus.

**Figure 2:**
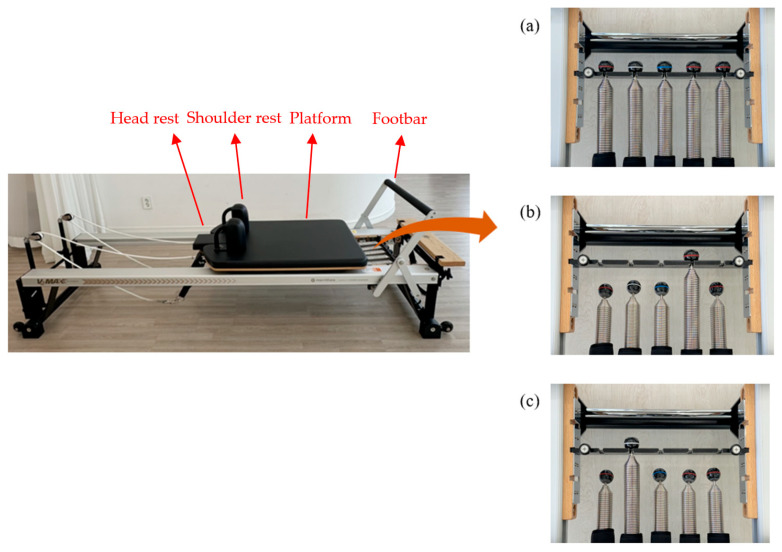
Pilates reformer andsprings: (**a**) fixed platform, (**b**) moderate resistance, (**c**) low resistance.

**Figure 3:**
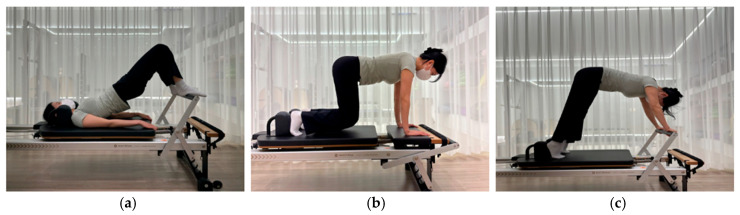
Three core muscle exercises: (**a**) hip roll, (**b**) knee-off, (**c**) elephant.

To test the effect of Reformer spring resistance conditions, the V2 Max (STOTT Pilates, Merrithew, Midtown, Toronto, ON, Canada) was used. The Reformer has a platform connected by springs, allowing for adjustment of exercise intensity through the application of the springs. The form and components of the Reformer are illustrated in Figure 2. During the experiment, the platform’s position was adjusted according to the manual of Merrithew’s ‘STOTT Pilates Reformer’. Three spring resistance conditions were created with a fixed platform and two unstable platforms connected by springs. The configuration of the Reformer’s springs consists of three red springs, one blue spring, and one white spring (Figure 2). The red spring has a resistance of 0.22 kg/cm, the blue spring has a tension of 0.11 kg/cm, and the white spring has a resistance of 0.06 kg/cm. All spring resistances were confirmed through the Reformer manufacturer [27]. The foot bar was adjusted to the appropriate height for each participant during the hip roll and elephant exercises, while it was moved outside the frame for the knee-off exercise. The headrest was positioned to support and align the neck with the spine. In addition, the shoulder rest was used to maintain shoulder alignment during the hip roll exercise while it was used as a foothold during the knee-off and elephant exercises (Figure 2 and Figure 3). A Pilates instructor (K.-H.J.) confirmed that there were no signs of spring damage, ensured the Reformer springs were securely attached, and checked the stability of the Reformer.

During Reformer exercises, different springs are typically set for each movement, but common exercises are often performed using just one red spring. This can be explained by the fact that a single red spring provides moderate resistance for Reformer exercises [27]. The three resistance spring conditions were fixed platform, moderate-resistance spring, and low-resistance spring settings. The fixed platform condition involved securing the platform in place by applying all the springs, ensuring no movement of the platform. The moderate-resistance spring condition involved connecting the platform using one red spring, while the low-resistance spring condition involved connecting the platform using one white spring.

Three core muscle exercises (a. hip roll, b. knee-off, c. elephant; Figure 3) were selected to highlight the characteristic focus on strengthening core muscles. These exercises are commonly used to improve core stability and are considered fundamental core exercises in Pilates [28,29,30,31]. Each exercise was performed according to Merrithew’s STOTT Pilates Reformer manual, with specific details as follows.

1. Hip roll

The participant lies supine on the Reformer’s platform with knees bent at a 90° angle, feet resting on the foot bar with heels supporting, and hands placed on the platform with palms facing downwards. The pelvis is positioned neutrally, and at the start of the exercise, it is tilted posteriorly to lift the torso from the coccyx to the thoracic spine. When the torso is lifted, the pelvis returns to a neutral position. As the torso is lowered, the pelvis is again tilted posteriorly, allowing the torso to descend sequentially from the thoracic spine to the coccyx, ultimately returning the pelvis to a neutral position on the platform. When exercising with the springs applied, caution should be taken to prevent the platform from sliding as the springs elongate.

2. Knee-off

The participant’s hands are positioned on this additional fixed platform, while the knees and legs remain on the original platform, assuming a quadruped position with the spine and pelvis in a neutral state. The knees are lifted approximately 10 cm off the ground, supported by the hands and toes, ensuring that the shape of the spine and pelvis remains unchanged.

3. Elephant

The elephant exercise involves creating flexion from the lumbar, thoracic, and cervical spine while maintaining a posterior tilt of the pelvis and pulling the lower body toward the torso. Hands are positioned on the foot bar at shoulder width, and heels are placed against the shoulder rest with heels touching to prepare. Starting with the pelvis in a neutral position, tilt it posteriorly. When applying the springs, push the platform back by 10 cm to create abdominal contraction, then bring the platform back to its original position.

### 2.3. Electromyography (EMG)

Surface EMG was recorded from six muscles: rectus abdominis (RA), internal obliques (IO), multifidus (MU), erector spinae (ES), rectus femoris (RF), and biceps femoris (BF). The Surface EMG is a non-invasive technique in which electrodes are placed on the skin to capture electrical signals of muscle activity during dynamic movements [32]. Due to the limited number of EMG electrodes, we included only the IO muscle, which is considered more involved in spinal stability than the external oblique [14,30]. Additionally, the transverse abdominis was not included since the Surface EMG is unable to assess deep core muscles. Prior to electrode placement, the skin was prepared by shaving excess hair and applying an abrasive paste (Ido Farm; Ido Skin Swab, Republic of Korea). Electrodes were positioned parallel to the muscle fibers following SENIAM guidelines, with all placements carried out by one researcher (J.-H.S.) to ensure consistency (Table 1) [33]. Improper skin preparation and inadequate sensor placement can lead to corrupted EMG data [33,34]. Raw EMG data were inspected for integrity through visual amplitude inspection and power spectral analysis. These signals underwent band-pass filtering using a fourth order Butterworth filter with a range of 10 to 450 Hz to reduce movement artifacts. Additionally, a notch filter set at 60 Hz with a fourth order Butterworth filter was applied to minimize interference from nearby electronic devices. The data were then rectified and processed with a low-pass filter at 10 Hz to obtain the EMG linear envelope. Amplitudes for individual muscles were calculated as the average of this linear envelope during core muscle exercises and normalized to the peak amplitude recorded during maximum voluntary isometric contraction (MVIC). All data analysis was conducted using customized MATLAB code.

### 2.4. Statistical Analysis

All statistical analyses were conducted using R version 4.3.2 (RStudio, Boston, MA, USA), with a significance threshold set at 0.05. This study employed a within-subject design, and thus, repeated measures of analyses of variance (ANOVA) were used to test the effect of Reformer spring resistance on core muscle activity. When significant main effects were found, Bonferroni post-hoc tests were performed. Normality was tested using the Shapiro−Wilk test. If the assumption of normality was violated, Friedman tests were employed instead of repeated measures ANOVA. When the main effect of the Friedman test was significant, Wilcoxon signed-rank tests were performed for pairwise comparisons. The effect size for repeated measures ANOVA was also calculated as the eta squared (η^2^) and considered small if η^2^ = 0.01, medium if η^2^ = 0.06, and large if η^2^ = 0.14 [35]. For Friedman tests, Kendall’s W values were also calculated as effect sizes, with W = 0.01, 0.06, and 0.14 corresponding to small, medium, and large effects, respectively [36]. The level of statistical significance for the repeated measures ANOVA was set at *p* < 0.05, while for the post-hoc tests, it was set at *p* < 0.0167 (0.05/3). To test the hypotheses, pairwise comparisons included differences between the three spring resistance conditions.

## 3. Results

There were significant differences in mean core muscle activity during the hip roll exercises. The mean muscle activity of ES, MF, RF, and BF was increased on the low-resistance spring platform compared to the fixed platform and moderate-resistance spring platform (*p* ≤ 0.002, Table 2). In addition, the mean RA muscle activity was increased on the low-resistance spring platform compared to the fixed platform. During the knee-off exercise, mean core muscle activity of RA, IO, ES, and MF was increased when comparing the low-resistance platform to the fixed platform (*p* ≤ 0.005, Table 3). Only mean RA muscle activity was increased on the low-resistance spring platform compared to the moderate-resistance spring platform (*p* < 0.001).

Lastly, during elephant exercise, the mean muscle activity of RA and RF was increased when comparing the low-resistance spring to the fixed and moderate spring conditions (*p* < 0.001, Table 4). Mean IO muscle activity was increased on the low- and moderate-resistance spring platforms compared to the fixed platform (*p* < 0.001).

## 4. Discussion

The purpose of this study was to investigate the effect of Reformer spring resistances on core muscle activity during core muscle exercises. Additionally, the stability of the exercise platform was manipulated using the Reformer spring resistance, which is needed to increase core muscle activity. Over 60% MVIC of core muscle activity could be more effective for strength development, while around 30% MVIC is sufficient for basic enhancement and rehabilitation [37,38]. Previous studies suggested that core muscle activity was less than 30% MVIC during low-intensity or therapeutic core exercises, indicating benefits for improving spine stability and regaining basic strength [38,39]. Another study also showed that patients with low back pain maintained low levels of IO and ES activity, reaching up to 28% of MVIC during bride exercise (comparable to hip roll) [30]. In this study, the highest mean EMG value was found in the IO muscle for the low-resistance spring platform (27% MVIC) during the elephant exercise. In this context, the core exercise method using Reformer spring resistance modification likely contributes more to core muscle stability and endurance improvement rather than strength enhancement [11,40]. Reformer core exercises could be effective and safe rehabilitation methods for individuals with spinal problems.

The Reformer is equipped with a platform connected to springs, allowing for the adjustment of exercise intensity by applying different levels of spring resistance [18,19]. We found that the low-resistance spring platform had a greater impact on core muscle activity compared to the fixed platform during the hip roll, knee-off, and elephant exercises. When comparing the low-resistance spring platform to the fixed platform during the hip roll exercise, RA increased by 28% (5.4% MVIC vs. 6.9% MVIC), ES increased by 54% (6.9% MVIC vs. 10.6% MVIC), and MU increased by 54% (8.9% MVIC vs. 13.7% MVIC). During the knee-off exercise, RA increased by 62% (11.2% MVIC vs. 18.1% MVIC), IO increased by 17% (17.9% MVIC vs. 21.0% MVIC), and ES increased by 22% (2.7% MVIC vs. 3.3% MVIC). Finally, during the elephant exercise, the RA showed a 228% (7.5% MVIC vs. 24.6% MVIC) increase, and the IO muscles showed a 66% (16.2% MVIC vs. 26.9% MVIC) increase. These findings suggest that core exercises on the unstable platform induced by the Reformer substantially increase core muscle activity. Similarly, a previous study reported that exercising on unstable ground can enhance overall balance, posture control, proprioception sense, and deep muscle activation [41,42]. When designing a Reformer exercise program aimed at core muscle endurance, it should start with static training and gradually decrease the spring resistance to effectively increase core muscle activity. It is also important to consider the Reformer spring resistance adjustment according to the appropriate level of core muscle strength for each individual in order to maintain core muscle stability. Therefore, our findings could be incorporated as objective evidence when structuring new core exercise programs using the Reformer.

Mean IO muscle activity was not sensitive to unstable platforms caused by the Reformer spring resistance during the hip roll and knee-off exercises. Conversely, a study found that mean IO muscle activity increased by 82% on an unstable surface compared to a stable surface during bridge exercise similar to hip roll [31]. Also, it was reported that bridge exercise on an exercise ball resulted in a 35% increase in mean IO muscle activity compared to on the floor [43]. The reason for the different results between our study and previous studies may be attributed to additional instability created by the Reformer springs attached to the platform. Instead, lower body muscle activity was increased on the low-resistance spring (unstable platform) conditions compared to the fixed platform condition (stable platform), which led to a 36% (2.5% MVIC vs. 3.4% MVIC) increase for RF and a 138% (6.9% MVIC vs. 16.4% MVIC) increase for BF. Accordingly, this could have led to unchanged IO muscle activity, indicating that participants tend to utilize low extremity muscles to maintain postures instead of core muscles. In this situation, during the hip roll exercise, the RF muscle is primarily involved in pushing the Pilates platform away, while the biceps femoris works to pull the sliding platform back.

During the hip roll exercise, the unstable platform caused by the low-resistance spring resulted in a 54% increase in ES muscle activity compared to the fixed platform. A previous study found no difference in ES muscle activity between bridge exercises performed on the floor and those performed on an exercise ball [43]. Both the hip roll and the bridge exercise studies require lifting the hips off the floor, resulting in spinal extension and hip extension. There is a positional difference of the feet between using an exercise ball and the floor, making it unclear how an unstable surface impacts core muscle activity. In this study, our experiment using the Reformer allowed us to create unstable surfaces while maintaining the same body positions through spring resistance settings. Therefore, our results indicate that the ES muscles play an important role in spinal stabilization on the unstable platform during trunk extension exercises.

There are several limitations of this study. One limitation is that Surface EMG may pose challenges for accurate measurement of deep core muscle activity. Multifidus EMG may be an indirect measure because the superficial muscles cover the surface of multifidus. Secondly, we did not include the transverse abdominis and external oblique muscles, which could also contribute to core muscle stability. However, the exclusion of these muscles from the analysis limits our understanding of the full spectrum of core muscle activity. While deep muscles (e.g., transverse abdominis) cannot be assessed using Surface EMG, needle and fine wire electrodes can be inserted directly into the muscle tissue to evaluate them [44]. Using the needle EMG approach, the transverse abdominis should be assessed to provide a complete picture of the effects of Reformer core muscle exercises. Thirdly, since this study solely relied on EMG for comparing muscle activity, so without additional video analysis, we are unable to know where in the body postural changes are being made. Lastly, we only recruited practitioners, not non-practitioners. Previous research has indicated that Pilates practitioners can maximize the activation of target muscles by performing precise movements [5]. Pilates practitioners could more effectively control their trunk muscles to provide stability, whereas non-practitioners may not do so. In addition, this study recruited only female participants, but there may be gender differences in core muscle activities under different Reformer spring resistances. This limits the generalizability of the results, as non-practitioners and males may have different core muscle activity patterns and postural control strategies. Therefore, further studies focusing on non-practitioners and male participants are needed to provide more comprehensive mechanisms of core muscle activity.

## 5. Conclusions

This study is the first that we know of to investigate the effect of Reformer spring resistance on core muscle activity during basic core muscle exercises. The unstable platform created by the low-resistance Reformer spring increased core muscle activity. Reformer exercises through different spring adjustments could be an effective method for core muscle training, particularly for core endurance and stability. Furthermore, when designing core muscle exercise programs using the Reformer, the appropriate level of platform stability should be considered as a key factor for rehabilitation and spine stability. Reformer core exercises may be an effective and safe rehabilitation method for individuals with spinal disorders and low back pain, when considering that core muscle activity is maintained at a low level, not exceeding 30% of MVIC.

## Figures and Tables

**Table 1:** Surface Electromyography sensor placement sites.

Muscle	Attachment Sites
Rectus abdominis	2 cm lateral and 2 cm superior to the umbilicus
Internal obliques	2 cm medial and inferior to the anterior superior iliac spine
Erector spinae	2 cm lateral to the spinous process of L1
Multifidus	3 cm lateral to the midpoint between the spinous processes of L1 and L5
Rectus femoris	Midway between the ASIS and the superior aspect of the knee
Biceps femoris	Midway between the ischium and the lateral epicondyle of the fibula

**Table 2:** Means and standard deviations for EMG amplitude during hip roll exercise.

	Fixed	Moderate	Low	Main Effect	
	% MVIC	% MVIC	% MVIC	*p*-Value	Effect Size (η^2^)
RA	5.4 (3.3)	6.3 (3.5) ^a^	6.9 (2.8) ^a^	*p* = 0.002	0.039
IO	19.1 (13.2)	19.6 (16.5)	18.3 (12.7)	*p* = 0.64	0.002
ES	6.9 (3.0)	8.5 (3.5) ^a^	10.6 (4.5) ^a,b^	*p* < 0.001 *	0.790 *
MU	8.9 (7.6)	11.1 (9.5) ^a^	13.7 (11.1) ^a,b^	*p* < 0.001 *	0.651 *
RF	2.5 (0.9)	2.9 (0.9) ^a^	3.4 (1.1) ^a,b^	*p* < 0.001	0.139
BF	6.9 (3.3)	11.6 (4.9) ^a^	16.4 (6.7) ^a,b^	*p* < 0.001	0.371

^a^ *p* < 0.0167 vs. fixed, ^b^ *p* < 0.0167 vs. moderate. * Friedman test and Kendall’s W value were applied. A larger effect size indicates more impactful findings, which are more likely to be relevant in real-world clinical or fitness scenarios [35].

**Table 3:** Means and standard deviations for EMG amplitude during knee-off exercise.

	Fixed	Moderate	Low	Main Effect	
	% MVIC	% MVIC	% MVIC	*p*-Value	Effect Size (η^2^*)*
RA	11.2 (5.6)	13.9 (5.6) ^a^	18.1 (7.0) ^a,b^	*p* < 0.001	0.188
IO	17.9 (8.0)	17.7 (7.7)	21.0 (9.4) ^a^	*p* = 0.004	0.032
ES	2.7 (1.1)	3.0 (1.2)	3.3 (1.3) ^a^	*p* = 0.005	0.033
MU	5.8 (8.4)	6.5 (8.8) ^a^	7.2 (9.6) ^a,b^	*p* < 0.001 *	0.481 *
RF	14.1 (6.7)	15.0 (6.5)	16.2 (9.8)	*p* = 0.183	0.012
BF	2.2 (1.2)	2.4 (1.5)	2.4 (1.4)	*p* = 0.092 *	0.133 *

^a^ *p* < 0.0167 vs. fixed, ^b^ *p* < 0.0167 vs. moderate. * Friedman test and Kendall’s W value were applied.

**Table 4:** Means and standard deviations for EMG amplitudes during elephant exercise.

	Fixed	Moderate	Low	Main Effect	
	% MVIC	% MVIC	% MVIC	*p*-Value	Main Effect (η^2^)
RA	7.5 (4.8)	14.4 (7.7) ^a^	24.6 (13.3) ^a,b^	*p* < 0.001	0.377
IO	16.2 (9.0)	23.1 (11.2) ^a^	26.9 (11.6) ^a^	*p* < 0.001	0.154
ES	3.3 (1.6)	3.1 (1.6)	3.5 (1.3) ^b^	*p* = 0.024 *	0.207 *
MU	5.6 (5.2)	5.7 (7.2)	6.8 (7.7) ^b^	*p* = 0.006 *	0.281 *
RF	8.0 (5.4)	12.3 (6.2) ^a^	16.0 (8.0) ^a,b^	*p* < 0.001	0.206
BF	2.9 (1.9)	2.9 (1.9)	3.3 (1.9)	*p* = 0.412	0.010

^a^ *p* < 0.0167 vs. fixed, ^b^ *p* < 0.0167 vs. moderate. * Friedman test and Kendall’s W value were applied.

## Data Availability

The data presented in this study are available on request from the corresponding author.
